# Supplementary material for: Projection in genomic analysis: A theoretical basis to rationalize tensor decomposition and principal component analysis as feature selection tools
Source: PLoS One. 2022 Sep 29;17(9):e0275472. doi: 10.1371/journal.pone.0275472 (PMC9521941; doi:10.1371/journal.pone.0275472)
Supplement: Attachment — Submitted filename: Comments-PONE-D-22-20332.pdf [file pone.0275472.s002.pdf]

Dear Editor:

Considering the manuscript with the manuscript: **PONE-D-22-20332**, entitled "**Projection in genomic analysis: A theoretical basis to rationalize tensor decomposition and principal component analysis as feature selection tools**", herewith I would like to submit my comments. This paper is well elucidated and well referenced, nevertheless, some more discussions need to be added to improve the comprehensiveness of demonstration of the method and some needs to be removed, revised or edited. Some minor issues need to be addressed or corrected to improve the general quality and readability of this article. The paper needs to be developed in some parts. So based on my comments that comes in the following, I proposed the paper to be subjected for moderate revision.

Best Regards

General Comments:

Is the paper new, technically correct, and relevant?

Yes, the paper is new and technically sounds. Results somehow does support the methodology, but needed to be more cleared by the author in case of properties of the data.

Is the paper well organized?

The paper is properly organized, good literature review, suitable motivation and clear explanation on results are positive points to that.

Is the abstract concise?

Yes, but I think it needs to be rephrased after revision to add some comments about any artifacts or negative points in the method, if exist.

Is the introduction motivating?

Yes, Introduction section is motivating.

Are the methodology, results, and conclusions completely developed?

No, they need to be modified and developed according to the technical comments.

Are there language, mathematics, reference, or style errors?
There is no mathematical, reference or style error.

Technical Comments:

Are the codes available for this research? As I found, there is no code available for this study, e. g. in Github. If the authors could make the codes available, the manuscript could be much better evaluated, not only for reviewers, but also for possible readers. When it is not possible to upload the code for public access, such as in Github, could they be provided for reviewer for better assessment of the study?

The study is comprehensive and requires large time to be read carefully and being reviewed. The theoretical background has been well explained in details, and the experiments and related models are presented and the algorithm in Fig. 1 is also well presented. I think more explanation about the steps and the parameters in Fig. 1 is required.

The result comparison parts are well organized and presented. The display way is good. But quantitative evaluation is somehow too much that one can get lost in that. I think it would be better that you add more explanation to that.

How did you evaluate the final result? How did you consider to finally selection a methodology for the most complicate problem?

What about when the models are more complex?

The introduction section is a nice one. It is architected very beautifully, while written fully academic and comprehend. I assume that any change in the introduction section is not necessary, but one of the important tasks after publishing a study is to increase its chance to be seen by the most possible number of researchers, so I would like to give two recommendations. First, to get your published study in the list of searched for papers based on keywords, I propose to increase variety of your keywords. In my viewpoint, they do not cover the whole topic of the study and are not widely searched words. I propose to add at least the keyword “data analysis”. Second, one of the methods in the publisher’s website that brings a publication on to the researchers, is based on the similar publications that they have read before. So, the more you cite similar publication, the more the chance that the search engine in the publisher website propose your paper to the researcher. Besides of that, it will also complete your introduction section. As another advantage, it rises new ideas to the researchers by combining various methods, or resolving drawback of one seen paper by reading the similar one, or extending the methodology to a fully automatic one. So, based on these points, I would like to ask to cite to the following similar publication in the manuscript which used PCA and feature selection for deep learning, but in different field of study. The first proposed publication is: Shahbazi, A., Soleimani Monfared, M., Thiruchelvam, V., Ka Fei, T., Babasafari, A.A., (2020). Integration of knowledge-based seismic inversion and sedimentological investigations for heterogeneous reservoir. Journal of Asian Earth Sciences. The second publication for citation is: Khayer, K., Kahoo, A.R., Soleimani Monfared, M., Tokhmechi, B., and Kavousi, K., (2022). Target-

Oriented Fusion of Attributes in Data Level for Salt Dome Geobody Delineation in Seismic Data. Natural resource research, and the other publication could be: Khayer, K., Kahoo, A.R., Soleimani Monfared, M., and Kavouosi, K., (2022). Combination of seismic attributes using graph-based methods to identify the salt dome boundary. Journal of Petroleum Science and Engineering. 215, Part A, 110625,

The abstract focusses mainly on the general problem and ignores the other items of the abstract such as the methodology, good introduction, results and conclusion.

The authors should explain what limitations did they find out about the proposed method.

Best Regard
