# Supplementary material for: Projection in genomic analysis: A theoretical basis to rationalize tensor decomposition and principal component analysis as feature selection tools
Source: PLoS One. 2022 Sep 29;17(9):e0275472. doi: 10.1371/journal.pone.0275472 (PMC9521941; doi:10.1371/journal.pone.0275472)
Supplement: Attachment — Submitted filename: PONE-D-22-20332_reviewer.docx [file pone.0275472.s001.docx]

The paper analyzes the reason why the recently proposed principal component analysis (PCA) and tensor decomposition (TD)-based unsupervised feature extraction (FE) has often outperformed these statistical test-based methods in the context of projection pursuit that was proposed a long time ago. Some findings in this paper rationalize the success of PCA- and TD-based unsupervised FE for the first time. I have the following suggestions for this manuscript.

1. The meaning of “$TrSb$” on page 11, line 1 cannot be thoroughly understood.
2. There are some formatting problems in the article that need to be corrected. For example,
(1) In the second paragraph on page 4, “(for more details about how PCA- and TD-based unsupervised FE are superior to statistical test-based feature selection tools in these specific examples, see these previous studies (Ng and Taguchi, 2020; Taguchi and Turki, 2020)” is a lake of the right bracket.
(2) In the last paragraph on page 4, the writing of Figure 1 is inconsistent with the rest of the paper.
(3) There is a lake of formula in (42) on page 11.
(4) The punctuation marks of formulas (20) and (36) are used incorrectly.
(5) Many formulas lack punctuation, such as (14), (29), (41), (45), and (46) etc. Please check carefully.
(6) Please pay attention to the font of “in silico” in the middle of page 12.
(7) On page 25, the annotation in Figure 3 is missing punctuation.
3. The whole article needs to be carefully examined to correct simple grammatical problems. For example,
(1) The clause of sentence, “Moreover, addition of L_1_ norm is known to be a poor strategy when $X$ are not composed of independent vectors” in line 6 on page 3, uses predicate verb incorrectly.
(2) In the last paragraph on page 3, a sentence uses two verbs, “Although the reason for the unpopularity of the projection strategy, eq. (7) is unclear, this may be explained by the ignorance of the contribution perpendicular to…”
(3) The sentences, “Instead of these two strategies, feature selection based on statistical tests (Fang et al., 2012; Chen et al., 2006) are more popular in genomic sciences as mentioned above.” and “Despite its popularity, feature selection based on statistical tests have critical problems;” in lines 2 and 5 on page 4, use predicate verbs incorrectly.
(4) In the last paragraph on page 6, a sentence uses two verbs, “where is a core tensor,,, are singular value matrices.”
(5) The sentence, “Next, K-means always identify the primary cluster.” in lines 9 on page 18, uses predicate verb incorrectly.
(6) On page 13, “and” should be added before “” of the sentence “where,,,.”
4. For the expression problems in this paper, it is suggested to use less “in order to”. In addition, “list” is used to describe the table and “show” is used to describe the figure.
5. Please check the references carefully. For example, reference [1], [3], [4], [5] and [7] etc.
