# Supplementary material for: Projection in genomic analysis: A theoretical basis to rationalize tensor decomposition and principal component analysis as feature selection tools
Source: PLoS One. 2022 Sep 29;17(9):e0275472. doi: 10.1371/journal.pone.0275472 (PMC9521941; doi:10.1371/journal.pone.0275472)
Supplement: Attachment — Submitted filename: Replies_to_reviewers.docx [file pone.0275472.s003.docx]

**Replies to reviewer 1**

Reviewer #1: The paper analyzes the reason why the recently proposed principal component analysis (PCA) and tensor decomposition (TD)-based unsupervised feature extraction (FE) has often outperformed these statistical test-based methods in the context of projection pursuit that was proposed a long time ago. Some findings in this paper rationalize the success of PCA- and TD-based unsupervised FE for the first time. I have the following suggestions for this manuscript. Other comments can see the attached file.

*>Thank you very much for your positive evaluations.*

1. The meaning of “$TrSb$” on page 11, line 1 cannot be thoroughly understood.

*>Thank you very much for your comments. It is a trace of matrix. We have denoted it explicitly in the paper.*

2. There are some formatting problems in the article that need to be corrected. For example,
(1) In the second paragraph on page 4, “(for more details about how PCA- and TD-based unsupervised FE are superior to statistical test-based feature selection tools in these specific examples, see these previous studies (Ng and Taguchi, 2020; Taguchi and Turki, 2020)” is a lake of the right bracket.
(2) In the last paragraph on page 4, the writing of Figure 1 is inconsistent with the rest of the paper.
(3) There is a lake of formula in (42) on page 11.
(4) The punctuation marks of formulas (20) and (36) are used incorrectly.
(5) Many formulas lack punctuation, such as (14), (29), (41), (45), and (46) etc. Please check carefully.
(6) Please pay attention to the font of “in silico” in the middle of page 12.
(7) On page 25, the annotation in Figure 3 is missing punctuation.

*>Thank you very much for your comments. We have corrected the above formatting problems.*

3. The whole article needs to be carefully examined to correct simple grammatical problems. For example,
(1) The clause of sentence, “Moreover, addition of L_1_ norm is known to be a poor strategy when $X$ are not composed of independent vectors” in line 6 on page 3, uses predicate verb incorrectly.
(2) In the last paragraph on page 3, a sentence uses two verbs, “Although the reason for the unpopularity of the projection strategy, eq. (7) is unclear, this may be explained by the ignorance of the contribution perpendicular to…”
(3) The sentences, “Instead of these two strategies, feature selection based on statistical tests (Fang et al., 2012; Chen et al., 2006) are more popular in genomic sciences as mentioned above.” and “Despite its popularity, feature selection based on statistical tests have critical problems;” in lines 2 and 5 on page 4, use predicate verbs incorrectly.
(4) In the last paragraph on page 6, a sentence uses two verbs, “where is a core tensor,,, are singular value matrices.”
(5) The sentence, “Next, K-means always identify the primary cluster.” in lines 9 on page 18, uses predicate verb incorrectly.
(6) On page 13, “and” should be added before “” of the sentence “where,,,.”

*>Thank you very much for your comments. We have corrected the above grammatical errors.*

4. For the expression problems in this paper, it is suggested to use less “in order to”. In addition, “list” is used to describe the table and “show” is used to describe the figure.

*>Thank you very much for your comments. We have corrected the above mistakes.*

5. Please check the references carefully. For example, reference [1], [3], [4], [5] and [7] etc.

*>Thank you very much for your comments. We have checked references.*

**Replies to reviewer 2**

Are the codes available for this research? As I found, there is no code available for this study, e. g. in Github. If the authors could make the codes available, the manuscript could be much better evaluated, not only for reviewers, but also for possible readers. When it is not possible to upload the code for public access, such as in Github, could they be provided for reviewer for better assessment of the study?

*>Thank you very much for your comments. We have uploaded sample R code that can perform analyses in this study at GitHub:* [*https://github.com/tagtag/peoj*](https://github.com/tagtag/peoj)

The study is comprehensive and requires large time to be read carefully and being reviewed. The

theoretical background has been well explained in details, and the experiments and related models are presented and the algorithm in Fig. 1 is also well presented. I think more explanation about the steps and the parameters in Fig. 1 is required.

*>Thank you very much for your comments. We have added more explanation in Fig.1 caption.*

The result comparison parts are well organized and presented. The display way is good. But quantitative evaluation is somehow too much that one can get lost in that. I think it would be better that you add more explanation to that.

*>Thank you very much for your comments. We have added some more explanations*

How did you evaluate the final result? How did you consider to finally selection a methodology for

the most complicate problem? What about when the models are more complex?

*>Thank you very much for your comments. We have added one paragraph at the end of discussion section.*

The introduction section is a nice one. It is architected very beautifully, while written fully academic and comprehend. I assume that any change in the introduction section is not necessary, but one of the important tasks after publishing a study is to increase its chance to be seen by the most possible number of researchers, so I would like to give two recommendations. First, to get your published study in the list of searched for papers based on keywords, I propose to increase variety of your keywords. In my viewpoint, they do not cover the whole topic of the study and are not widely searched words. I propose to add at least the keyword “data analysis”. Second, one of the methods in the publisher’s website that brings a publication on to the researchers, is based on the similar publications that they have read before. So, the more you cite similar publication, the more the chance that the search engine in the publisher website propose your paper to the researcher. Besides of that, it will also complete your introduction section. As another advantage, it rises new ideas to the researchers by combining various methods, or resolving drawback of one seen paper by reading the similar one, or extending the methodology to a fully automatic one. So, based on these points, I would like to ask to cite to the following similar publication in the manuscript which used PCA and feature selection for deep learning, but in different field of study. The first proposed publication is: Shahbazi, A., Soleimani Monfared, M., Thiruchelvam, V., Ka Fei, T., Babasafari, A.A., (2020). Integration of knowledge-based seismic inversion and sedimentological investigations for heterogeneous reservoir. Journal of Asian Earth Sciences. The second publication for citation is: Khayer, K., Kahoo, A.R., Soleimani Monfared, M., Tokhmechi, B., and Kavousi, K., (2022). Target-Oriented Fusion of Attributes in Data Level for Salt Dome Geobody Delineation in Seismic Data. Natural resource research, and the other publication could be: Khayer, K., Kahoo, A.R., Soleimani Monfared, M., and Kavouosi, K., (2022). Combination of seismic attributes using graph-based methods to identify the salt dome boundary. Journal of Petroleum Science and Engineering. 215, Part A, 110625,

*>Thank you very much for your comments. We have added keyword and references.*

The abstract focusses mainly on the general problem and ignores the other items of the abstract such as the methodology, good introduction, results and conclusion.

*>Thank you very much for your commnts. We have added more sentences in abstract.*

The authors should explain what limitations did they find out about the proposed method.

*>Thank you very much for your comments. We have added one paragraph at the end of discussion section.*
